# Nursing care in potential organ donor maintenance: EMPDO website validity

**DOI:** 10.1590/0034-7167-2024-0515

**Published:** 2025-12-08

**Authors:** Cláudia Moura Santiago, Cleisiane Xavier Diniz, Maria de Nazaré de Souza Ribeiro, Tereza Cristina Felippe Guimarães, Amélia Nunes Sicsú, Fátima Helena Espírito Santo

**Affiliations:** I Universidade do Estado do Amazonas. Manaus, Amazonas, Brazil; II Instituto Nacional de Cardiologia. Rio de Janeiro, Rio de Janeiro, Brazil; III Universidade Federal Fluminense. Niterói, Rio de Janeiro, Brazil

**Keywords:** Nursing Care, Organ Donation, Transplant, Biomedical Technology, Web Site., Atención de Enfermería, Donación de Órganos, Trasplante, Tecnología Biomédica, Sitio Web.

## Abstract

**Objectives:**

to validate care-educational technology, such as a website, with the aim of supporting nursing care in potential brain-dead donor maintenance.

**Methods:**

a methodological approach study for technical-scientific and didactic-illustrative validity of EMPDO website. In the technical-scientific dimension, nurses assessed the “objectives”, “relevance”, “structure and presentation” domains, using the Content Validity Index. In the didactic-illustrative dimension, web designers assessed the “content”, “language”, “graphic illustrations”, “cultural adequacy” and “motivation” domains. The Suitability Assessment of Materials cut-off point was used.

**Results:**

the overall Content Validity Index of the technical-scientific content reached 0.97. In the didactic-illustrative dimension, 25.7 points out of 26 points were achieved. Suggestions allowed inconsistencies to be corrected for the final version.

**Conclusions:**

the website, built with the best scientific evidence of nursing care in potential brain-dead donor maintenance, received a favorable opinion from experts, who emphasized that it is an innovative, unprecedented and relevant technology.

## RESUMEN

**Objetivos:**

validar una tecnología de atención educativa, similar a un sitio web, para apoyar la atención de enfermería en el mantenimiento de posibles donantes de órganos en muerte cerebral.

**Métodos:**

estudio de enfoque metodológico para la validación técnico-científica y didáctica-ilustrativa del sitio web de EMPDO. En la dimensión técnico-científica, las enfermeras evaluaron los dominios “objetivos”, “relevancia”, “estructura y presentación”, utilizando el Índice de Validez de Contenido. En la dimensión didáctico-ilustrativa, los diseñadores *web* evaluaron los dominios “contenido”, “idioma”, “ilustraciones gráficas”, “idoneidad cultural” y “motivación”. Se utilizó el punto de corte de la *Suitability Assessment of Materials*.

**Resultados:**

el Índice de Validez de Contenido general del contenido técnico-científico fue de 0,97. En la dimensión didáctico-ilustrativa, se obtuvieron 25,7 de 26 puntos. Las sugerencias permitieron corregir inconsistencias en la versión final.

**Conclusiones:**

el sitio *web*, construido con la mejor evidencia científica de los cuidados de enfermería en el mantenimiento de potenciales donantes en muerte cerebral, recibió una opinión favorable de los expertos, quienes destacaron que se trata de una tecnología innovadora, inédita y relevante.

## INTRODUCTION

Organ donation and transplant processes have been undergoing transformations and scientific advances that seek to promote improvements in individuals’ quality and life prospects, through training of the responsible health team and uPODates based on current and innovative scientific evidence^(1)^.

Despite technical and scientific progress and standardization of care for potential organ donors (POD) in brain death (BD), there are still obstacles that result in fragmented care. Some of these include the team’s lack of preparation in family interviews, the lack of theoretical and practical knowledge in patient hemodynamic maintenance, failure to implement or delay in opening the BD protocol in the units, and lack of infrastructure in hospitals^(2)^.

Nurses play a very important role in the organ donation and transplant team; therefore, they need to be qualified and trained for various tasks, including donor identification, notification to the intra-hospital donation coordination team, donor monitoring and maintenance. Also noteworthy is the welcoming and empathy with the donor’s family, which will directly impact the effectiveness of organ procurement^(1,3-6)^.

By becoming part of organ transplant culture, nurses come to understand the process of caring for POD, in care management, which begins with welcoming the family, maintaining POD and referring the eligible donor, ending with the return of the body to the family^(5)^, making nurses the main facilitators of the organ donation process. Their knowledge and attitude towards organ donation can influence public and family opinion about organ donation^(6)^. In this regard, the study results showed that 69% of organ donations for transplantation occurred through nurse interventions. Factors such as religion, education, work experience, age, relationship with the deceased’s family, history of caring for brain-dead patients, and personal experiences impact nurses’ attitudes toward organ donation^(7)^.

In Brazil, researchers highlight the importance of nurses for the success of the organ donation process, but also identify several discrepancies in terms of knowledge and feelings that arise in the care of POD^(8,9)^. Some nurses, when dealing with POD with BD, identify the importance of maintaining care, but others claim not to prioritize POD with BD because they do not have vital viability. Thus, they consider it more important to care for those with a life prognosis, neglecting the necessary care for POD maintenance^(9)^.

A cross-sectional study conducted with 377 healthcare professionals in Tehran^(10)^ showed that willingness and reluctance to donate organs were 47.48% (n=179) and 52.51% (n=198), respectively, among these professionals. In Ireland, nurses reported not being aware of the organ donation process and BD diagnosis^(11)^. In South Korea, in a survey conducted with nursing students, 85.1% reported not having received any training on organ donation in BD; 68% were willing to donate their organs if they had BD; and 39.5% were willing to donate the organs of family members under these conditions^(12)^. In India, less than half of healthcare students who participated in a survey had adequate knowledge about BD and organ donation legislation^(4)^.

There is a certain barrier to confronting the idea of finitude while immersed in everyday activities^(13)^. This may be one of the main reasons why the topic of organ donation is not on the agenda of discussions in society, while statistics continue to show a considerable loss of PODs worldwide^(13)^. Added to this are the various issues related to the fragility of the health team and the maintenance of POD with BD, highlighting the essential need to develop educational strategies aimed at the health team, especially the nursing team^(1)^.

In this regard, technological advances have favored healthcare improvement in an effective and safe manner. Health technologies have expressed new ways of producing them, anchored in creativity and developed based on demands of reality^(14)^. Educational resources in digital format such as websites are important tools in the teaching-learning process, as they help to improve knowledge of situations experienced at opportune moments^(15)^.

Information and Communication Technologies (ICTs) contribute to scientific and technological development in the health area, providing agility in information processing and expanding the interaction between professionals and users^(16)^, promoting technological solutions that support clinical decision-making, interactions and qualified therapeutic conduct^(17)^. Considering the several advantages of ICTs, a care-educational technology, called EMPDO website, was built to guide nursing care in maintaining POD with BD.

EMPDO website was launched in 2023 and consists of a care-educational technology developed within the scope of the professional master’s program in public health nursing at the *Universidade do Estado do Amazonas*. With the identification of the best technical, scientific and normative evidence related to nursing care in the maintenance of POD with BD, based on a scoping review, adding to this researchers’ professional practice, the contents were listed, organized on the following topics that made up the website: Home; About EMPDO; Organ donation; Care practices in POD care; Exclusive materials; Legislation; Bulletins; and News. The tool is available at the electronic address https://www.EMPDO.com.br, and can also be accessed via a QR Code.

EMPDO website was created with scientific rigor and a close look at practices related to organ donation, with the aim of promoting holistic care, aiming for favorable results in POD preservation in a state of BD. However, a technology, in order to be applicable, needs to be validated as to its efficiency and applicability, proving whether it fulfills the purpose for which it was created^(18,19)^.

## OBJECTIVES

To validate care-educational technology, of the website type, with the aim of supporting nursing care in maintaining POD with BD.

## METHODS

### Ethical aspects

The research was approved by the *Universidade do Estado do Amazonas* Research Ethics Committee. To guarantee anonymity of participating experts, when referenced in the text, all received a letter and a number in reference to the category, followed by the chronological order of response to the form: Nurse (N1, …); Designer (D1, …). All signed the Informed Consent Form (ICF).

### Study design, period and location

This is a study of methodological approach and technological production, which corresponded to the validity, by health experts (nurses) and web designers, of a website called EMPDO website.

To define the sample size of expert nurses who validated the technical-scientific dimension, the formula proposed by Interaminense (2020) was used: n = Zα2. P (1-P)/d2. Where: Zα2 = 1.96; P = 0.85; d = 0.15. The result was a total of 22 experts, a number considered adequate for an assessment with an ideal proportion of 85% acceptance among evaluators, with a minimum proportion of 70%, considering a 95% Confidence Interval^(20)^.

In order to reach the appropriate number of sample subjects, a number of 30 experts was sought, maintaining a safety margin of eight additional experts. Among the 30 who received the invitation, 28 assessed and validated the proposed technology content.

For the sample of experts in the didactic-illustrative dimension (graphic designer/web designer), seven experts were selected. Research indicates that the ideal average is between three and five participants^(21-23)^.

### Population or sample; inclusion and exclusion criteria

The selection of all experts was carried out in a non-probabilistic and intentional manner, by consulting the resumes available on the *Lattes* Platform, using the subject search tool, in accordance with the determined inclusion criteria.

The health experts, nurses, included in the research, needed to meet at least two of the inclusion criteria: have at least three years of experience in Intensive Care Unit and POD maintenance or related areas; have publication in the area of transplantation and/or POD maintenance; have a master’s or doctoral degree with scientific production in intensive care or production of care-educational technologies or in related areas; have been part of a multidisciplinary organ transplant team for at least one year.

Concerning experts in the didactic-illustrative dimension (graphic designer/web designer), two of the following criteria were required: professional experience with the format/modality of care-educational technologies for at least two years; have at least three years of experience in their area of activity; have studies in the area of care-educational technologies; be a expert *lato* or *stricto sensu* in the professional area of web designer or graphic designer.

As exclusion criteria for all, those who remained without responding until the 15^th^ day after accepting the invitation, communicated to the project coordination any manifestation of psychological distress during the assessment process and presented difficulties in understanding the method were considered ineligible.

Experts from both areas received an invitation letter via electronic contact (email via the *Lattes* Platform). They were then sent the link to Google Forms^®^, the link to the assessment website, the specific ICF for each panel of evaluators, the assessment instruments and the necessary information regarding the completion of documents.

### Study protocol and analysis of results

To collect data for assessing the technical-scientific dimension, the parameters of the adapted validity instrument were used, consisting of three domains (objectives, relevance, structure and presentation) and 17 items with a score from 1 to 4, as follows: 4 - totally adequate; 3 - adequate; 2 - partially adequate; and 1 - inadequate. Space was added for experts’ suggestions. For these data, the Content Validity Index (CVI) was applied, which assesses experts’ agreement regarding the representativeness of the measure in relation to the content addressed^(24,25)^.

For the calculation by domain, the items assessed with totally adequate and adequate were added together, divided by the total number of responses in the domain. The overall value was obtained with the arithmetic mean of the CVI per domain, with a minimum validity parameter by the CVI of 0.70^(23,26)^. The percentage of agreement that validates an instrument can vary from 70% to 100%^(27)^.

In collecting data from the didactic-illustrative dimension, the instrument used was the Suitability Assessment of Materials (SAM), adapted from Galdino (2014), which assesses technology in five domains: content; language; graphic illustrations; cultural adequacy; and motivation^(28)^. It has 13 assessment items, with a value from 0 to 2 (0 - inadequate; 1 - partially adequate; and 2 - adequate), to obtain a score equal to or greater than 10 points out of a total of 26 points^(23,29)^.

The data were entered into Microsoft Excel^®^ spreadsheets, double-checked to avoid errors in inputting the analysis database, followed by transfer to the Statistical Package for the Social Sciences^®^ version 24.0 statistical software. Absolute and relative frequencies and averages were calculated.

All the notes, opinions and suggestions described by experts in the comments spaces during technology assessment were analyzed. This stage allowed us to identify inconsistencies in relation to the website content and define the content for the second version.

## RESULTS

Concerning the profile of experts who assessed the technical-scientific dimension, the majority were women (n = 23; 82%), with more than three years of experience in the area (60.7%) and an average of 15.75 years; 13 (46.4%) had *stricto sensu* degrees (doctoral and master’s degrees); and 14.2% had scientific production on the topic of interest or participation in events on the topic in the last five years, respectively.

Table 1 presents questions about the website content with their respective weighted averages (CVI per domain). It is worth noting that, to evaluate each item, the parameters of the adapted validity instrument were used, consisting of three domains (objectives; structure and presentation; and relevance). The analysis of responses was performed by calculating the CVI per domain and, subsequently, by the overall value. The validity parameter was 0.70 CVI.

**Table 1:** Distribution of agreement scores and Content Validity Index according to items by domain obtained from responses of expert judges of the technical-scientific dimension of EMPDO website, 2023

1 - OBJECTIVES - They refer to purposes, goals or ends that one wishes to achieve through the use of technology.							
**Domain items**		**TA**	**A**	**PA**	**I**	**TA+A**	**CVI**
1	The information and/or content is or is consistent with the daily needs of the technology’s target audience.	15	13	0	0	28	1.0
2	The information and/or content is important for quality of care or the work of the technology’s target audience.	15	13	0	0	28	1.0
3	It invites and/or instigates changes in care in potential organ donor maintenance by nurses.	11	16	1	0	27	0.96
4	It can circulate in the scientific community of the area (health).	19	9	0	0	28	1.0
5	It meets the objectives of research and institutions that serve/work with the technology’s target audience.	16	12	0	0	28	1.0
**CVI domain 1 (objectives): 0.99**							
**2- STRUCTURE AND PRESENTATION - Refers to the way in which the guidelines are presented. This includes overall organization, structure, presentation strategy, coherence, and formatting.**							
**Domain items**		**TA**	**A**	**PA**	**I**	**TA+A**	**CVI**
1	The technology is appropriate for the target audience.	13	15	0	0	28	1.0
2	The content is presented in a clear and objective manner.	16	11	1	0	27	0.96
3	The information presented is scientifically correct.	14	13	1	0	27	0.96
4	The material is appropriate for the target audience.	9	18	1	0	27	0.96
5	There is a logical sequence of the proposed content.	14	14	0	0	28	1.0
6	The information is well structured in terms of agreement and spelling.	11	16	1	0	27	0.96
7	The text is clearly described and/or summarized, responding to the target audience’s needs.	10	16	2	0	26	0.92
**CVI domain 2 (structure and presentation): 0.96**							
**3 RELEVANCE - Refers to the characteristics that assess the degree of significance of the technology.**							
**Domain items**		**TA**	**A**	**PA**	**I**	**TA+A**	**CVI**
1	The topics portray key aspects that should be reinforced.	15	13	0	0	28	1.0
2	Technology allows for the generalization and transfer of learning to different contexts.	12	15	1	0	27	0.96
3	Technology proposes knowledge construction.	17	11	0	0	28	1.0
4	Technology addresses the subjects necessary for the target audience to know.	12	16	0	0	28	1.0
5	Technology is suitable for use by any professional with the target audience.	15	12	1	0	27	0.96
**CVI domain 3 (relevance): 0.98**							
Domain 1 - objectives/domain 2 - structure and presentation/domain 3 - relevance **OVERALL CVI:** domain 1 (0.99) + domain 2 (0.96) + domain 3 (0.98) ---------------------------------------------------------------------------------------------------/3 = **0.97**							

*CVI - Content Validity Index; TA - totally adequate; A - adequate; PA - partially adequate; I - inadequate.*

Chart 1 summarizes the expert assessments of EMPDO website, covering the “objectives”, “structure and presentation” and “relevance” domains. In the observations tab, some suggestions were made by the research participants, which were analyzed and accepted, in order to improve the website’s content and visualization.

**Chart 1:** Considerations of nursing experts regarding the “objectives”, “structure and presentation” and “relevance” domains, and responses of researchers about EMPDO website

Domain objectives	
**Expert considerations**	**Researcher’s considerations**
N2, N3, N10, N25 - Suggest reviewing the Nursing Care Systematization, as some diagnoses do not fit and there are texts that were not mentioned in the Nursing Process (stages).	The Nursing Process was adjusted with all the stages described and listed (data collection, diagnoses (NANDA)/planning (NOC)/interventions (NIC), assessment, other care and nursing strategies).
N3, N9, N11, N20 - They expect the website to be updated annually.	Biannual updates are scheduled with the help of teachers and students in partnership with the *Universidade do Estado do Amazonas*.
N16 - Suggests a change in the nomenclature and acronym for potential organ donor to POD.	The acronym was corrected to POD - potential organ donor.
N12, N21, N22, N28 - Suggest adding suggestions, comments and films.	Two pages were created to provide opportunities for comments and suggestions, films and more.
**Domain structure and presentation**	
**Expert considerations**	**Researcher’s considerations**
N2 - Suggests, on the main page, rectifying the numeral agreement of “nurses”.	Fixed, as pointed out by N2.
N5, N6, N10 - Suggest that the text be revised in Portuguese, with spelling, formatting and accentuation checked.	The text has been revised.
N14 - Suggests that it would be interesting to add an “algorithm for managing potential donor patients”.	Some highlighted algorithms that are already being used have been added.
N16 - Suggests creating a QR Code for advertising.	A QR Code has been created for dissemination.
N15 - Suggests changing it to “there are two types of donors: living or deceased (corpses)”.	The word “corpses” has been added.
N17, N12, N13, N18, N20 - Suggests events to promote the website and for professionals specialized in the area.	A dissemination agenda has been created for events related to the nursing field.
N28 - Suggests that cornea care needs to be reviewed and better explained.	Content has been added and improved on the care related to cornea donation.
N21 - Suggests writing articles and registering (inserting) the website.	The suggestion has been accepted, with the inclusion of several articles on the subject on the website. The articles prepared by the authors will be included on the website after their due publication.
**Domain relevance**	
**Expert considerations**	**Researcher’s considerations**
N6 - Suggests that, due to its relevance, it could have a tab to access the schedule of national and/or international events/courses, and contact details for State Transplant Centers.	The State Transplant Centers contacts have been added and events related to the topic will be added to the website.
N10 - Suggests that, on some pages, the images are not clear. In order to be relevant, they need to be improved.	The images have been improved in terms of their sharpness.

### Validity of the didactic-illustrative dimension/appearance of EMPDO website by graphic designers/web designers

Seven web designers responded to the didactic-illustrative/appearance dimension assessment. Each one received a fictitious identification, represented by the letter D (designer) and numbers from 1 to 7, in order to protect their identity. Among those who assessed the technology, 57% were men; 57% had *stricto sensu* degrees; 100% had graduated for more than seven years; all had more than four years of experience.

To assess the didactic-illustrative/appearance dimension, SAM was used, with five dimensions, namely “content”, “language”, “graphic illustrations”, “motivation” and “cultural adequacy”, with a total of 13 items, with a minimum cut-off point of 10 points and a maximum of 26 points, with a value from 0 to 2 for each item, being: 2 - if they agreed that the website is adequate; 1 - partially adequate; and 0 - inadequate^(23,29)^.

The overall score reached 25.7 points, with a maximum score of 26 points, as judged by web designers/graphic designers as adequate. The assessment ranged from 25 to 26 points, with two experts assessing the material with 25 points (Chart 2).

**Chart 2:** Individual scores of web designers by domain obtained from responses on the technical-scientific dimension of EMPDO website, 2023

EXPERTS	SCORING BY DOMAIN					
	Content	Language	Graphic illustration	Motivation	Cultural suitability	Final score
D1	6	5	4	6	4	25
D2	6	6	4	6	4	26
D3	6	6	4	6	4	26
D4	6	6	3	6	4	25
D5	6	6	4	6	4	26
D6	6	6	4	6	4	26
D7	6	6	4	6	4	26
**Mean score**		Score by experts/total experts 25+26+26+25+26+26+26 / 7 **Mean score = 25.7 points**				

Concerning the score assigned by domain, Chart 3 shows that the “content”, “motivation” and “cultural suitability” domains obtained maximum scores in all their assessed items, reaching 100%. The overall calculation was made by multiplying the responses of seven experts, reaching 180 points (98.9%) out of a total of 182 points (100%) (Chart 3).

**Chart 3:** Distribution of points with each web designer according to responses obtained in each item according to content, language, graphic illustrations, motivation and cultural adequacy of EMPDO website, 2023

Domain items		A 02	PA 01	I 00	Points obtained
**Domain 1 - CONTENT**					
1.1	The objective is clear, facilitating the immediate understanding of the website.	07	-	-	14
1.2	The content covers information related to nursing care in potential organ donor maintenance.	07	-	-	14
1.3	The website’s proposal is interesting and innovative, allowing the target audience to access content on the topic in question.	07	-	-	14
	**Domain 1 - Content**	**42**			
**Domain 2 - LANGUAGE points**					
2.1	The reading level is appropriate for the target audience (healthcare professionals) to understand.	07	-	-	14
2.2	The visual style is consistent with the content described.	06	-	-	13
2.3	The vocabulary uses common words.	07	-	-	14
	**Domain 2 - Language**	**41**			
**Domain 3 - GRAPHIC ILLUSTRATIONS**					
3.1	The interface attracts attention and portrays the website’s purpose.	07	-	-	14
3.2	The visual illustrations and descriptions present key messages so that the reader can understand the care needed to maintain potential organ donors.	06	01	-	13
	**Domain 3 - Graphic illustrations**	**27**			
**Domain 4 - MOTIVATION**					
4.1	The text interacts with the reader, leading them to make choices to maintain potential organ donors.	07	-	-	14
4.2	There is a logical sequence of the proposed content, addressing the topic in question.	07	-	-	14
4.3	The website is appropriate for the target audience.	07	-	-	14
	**Domain 4 - Motivation**	**42**			
**Domain 5 - CULTURAL ADAPTATION**					
5.1	The website can be used by professionals from any region.	07	-	-	14
5.2	It presents appropriate images and examples.	07	-	-	14
	**Domain 5 - Cultural suitability**	**28**			
**OVERALL SCORE**		**42 + 41 + 27 + 42 + 28 = 180 points** **Seven experts** **25.7 points (98.9%)**			

*A - adequate; PA - partially adequate; I - inadequate.*

**Figure 1:**
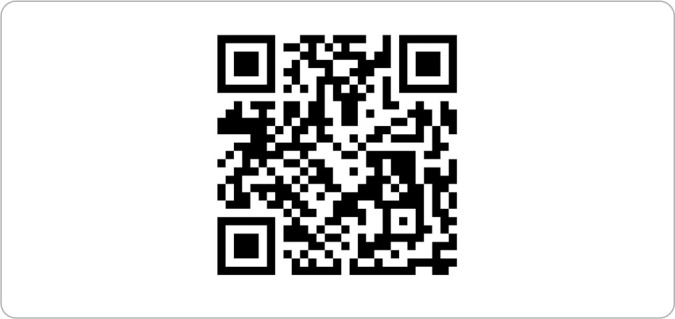
EMPDO website QR Code

The “EMPDO - Nursing in the Maintenance of Potential Organ Donors” website can be accessed directly via the link www.EMPDO.com.br or through a QR Code (Figure 1). The platform offers a series of external resources and guidelines for nursing practice in POD maintenance, providing updated information, technical manuals, explanatory videos and guidelines based on scientific evidence. Moreover, the website presents interactive descriptions for consulting protocols and accessing exclusive materials, facilitating the improvement of technical knowledge and support for care for nursing practices in POD.

## DISCUSSION

Domain 1 (objectives) has five items and refers to the purposes, goals or ends that one wishes to achieve with the use of technology. Of the 28 experts, 27 (96.4%) judged the items in this domain as totally adequate and adequate, and only one judged one of the items as partially adequate. Table 1 shows that there was agreement, with an CVI value of 0.99 for objectives, of the CVI among all domains, validating the website in this item.

In domain 2 (structure and presentation), the website organization, structure, presentation strategy, coherence and formatting were assessed in the seven items present in the instrument. Table 1 shows that 26 (92.8%) of experts assessed the technology in these items as totally adequate and adequate. It is noteworthy that, in some items, assessment reached 100% of responses for totally adequate and adequate. The CVI of this domain reached a value of 0.96. Despite having reached a value considered high for acceptance, there were six responses indicating partially adequate, with notes directed at spelling errors.

In domain 3 (relevance), the degree of significance of the technology was assessed in five items, in which 27 (96.4%) of experts rated it as totally adequate and partially adequate, with agreement on most items. There was a “partially adequate” assessment in only two items, reaching a CVI of 0.98.

When nursing experts assessed the website content, all the questions asked achieved a CVI above 0.90. Therefore, there was no need to make any major changes.

Regarding the “objectives” domain, N1, N5 and N14 mentioned that the website has an excellent presentation and content, that the material is very well prepared and meets the proposed objectives and goals. N8 mentioned that “the technology is innovative and will facilitate the understanding of professionals through access to the content”. N7, N23 and N24 highlighted the website’s relevance and excellence and that it will contribute to the improvement of healthcare professionals and will assist in the routine of those who work with POD of an agency.

As for the “structure and presentation” domain, N1, N4, N7, N9, N14, N23 and N24 highlighted the website’s excellence in terms of structure and presentation, congratulating the authors for the content included, presented in a clear, objective, well-described and/or summarized manner, responding to the target audience’s needs, with content in the form of well-prepared text. The evaluators considered it to be an accessible, free and universal technology for nursing professionals. N8 highlighted that “it is a technology that fits the proposal of disseminating more knowledge to the general public, as there is still a lot of resistance to organ donation due to lack of knowledge”.

In the “relevance” domain, N1, N14, N23 and N24 described the website as an important and excellent tool, with a clear method for use by health professionals. N6 highlighted that “the tool is very relevant for improving the care provided”. N18 emphasized that “it is a technology that will help disseminate more knowledge and allow generalization and transfer of learning in different contexts”. N21 stated that “it is in the appropriate form to be used by any nursing professional and, therefore, is highly relevant”.

### Validity of the didactic-illustrative dimension/appearance of EMPDO website by graphic designers/web designers

Experts did not make any comments or suggestions; therefore, the website did not require any changes in this regard. The website was considered adequate and can be used as a strategy to disseminate knowledge and improve nurses’ techniques and skills in organ donation and transplantation.

Studies show that the development of educational and technological strategies focused on professional qualification and training, aiming at quality of care, is a necessity in the current nursing scenario^(15,30)^.

Therefore, it is observed that knowledge is fundamental for POD care so that there is no deficit and gaps in this care, standardization and use of protocols and assistance guides in the care to be provided must be sought^(1,31)^. However, studies show that there is still a weakness in the provision of care in the process of organ donation and transplantation. The nursing team commonly demonstrates insecurity about the support to be given to POD and doubts about the ethical issues involving these patients^(1,32)^.

In this context, EMPDO website was produced with scientific and in-depth knowledge of POD care, with the aim of providing comprehensive care, aiming at positive results in maintaining POD with BD. By accessing EMPDO website, the user will find various scientific contents related to POD maintenance.

To ensure the Nursing Process efficiency and effectiveness, emphasis was placed on the care of patients with BD, emphasizing the importance of a thorough clinical and social assessment. Among the interventions, the following stood out: care with continuous hydration of the corneas, monitoring of vital signs, performance of electrolyte and blood gas measurements, blood cultures and urine cultures, and verification of responses in relation to expected results.

The website took approximately four months to build, from content development to finalizing the site structure, totaling 49 web pages. It is implemented with a registered domain. Within what is expected of a care-educational technology, EMPDO website encourages the sharing of relevant information that contributes to nursing care for the maintenance of POD with BD. More than its original proposal and concept, it is always important to understand the positive impact it can have on those who use it.

### Study limitations

As limitations of this study, the limited time for application and assessment of the website’s usability stands out.

One of the points of divergence between different authors is the number and qualifications of experts involved in the validity process. Some suggest a participation ranging from five to ten people in this process; others indicate a range of six to twenty participants. When making this decision, it is essential to consider the instrument characteristics, as well as the training, competency and availability of the professionals required. The sample of 28 health experts determined for this study caused a delay in reaching the desired number, which can be reconsidered for future studies using a quantitatively smaller and qualitatively better sample.

Although SAM is considered a useful tool for assessing the educational material suitability, it has significant limitations; among them, it is considered that the assessment is susceptible to subjective influences of the evaluator, which can lead to variations in the results. Personalization for the target audience and understanding of the data presented are vital for the effectiveness of materials, especially in health and education. Therefore, despite its relevance, it is essential to consider these limitations of SAM and seek complementary approaches for more accurate assessments.

In relation to the website, the need to continually keep up with the rapid advancement of technology and adjust the tool in use requires constant investment and updating. Connection difficulties and other technical issues can impact the platform’s performance and lead to its disuse.

### Contributions to nursing, health or public policy

Within what is expected of a care-educational technology, EMPDO website encourages the socialization of relevant information that contributes to nursing care for the maintenance of POD with BD.

Interactive resources, such as EMPDO website, make learning more dynamic and engaging, enhancing communication and research skills that are essential in today’s world. The content provided can be constantly updated, ensuring that all interested parties have access to the latest information in the area in question, promoting nurses’ engagement in POD care.

## CONCLUSIONS

In this study, multidisciplinary validity involving nurses and graphic designers was adopted. The former validated the technical-scientific content, and the latter, the didactic-illustrative/appearance dimension. In both categories, the technology produced obtained a satisfactory assessment, corroborating to ratify its relevance regarding what is proposed.

In the distribution of agreement scores and CVI, according to the items per domain obtained from the responses of expert nurses who assessed the technical-scientific dimension of EMPDO website, the result obtained by calculating the CVI per domain was domain 1 (0.99), domain 2 (0.96), domain 3 (0.98). The overall CVI was 0.97.

Regarding the responses obtained from web designers, who assessed content, language, graphic illustrations, motivation and cultural suitability, the overall score reached 25.7 points, out of a maximum of 26 points, which accounts for 98.9%.

The EMPDO website validity achieved a score above 0.90 in the specific and overall CVI regarding the technical-scientific content and in the didactic-illustrative/appearance dimension, which means that the product was considered adequate, receiving a favorable opinion from nursing experts and web designers, who emphasized that it is an innovative, unprecedented and highly relevant technology.

Suggestions, although specific, were accepted, improving the care-educational technology. It is expected that EMPDO website will contribute theoretically and practically to nursing professionals working in the organ transplant and donation process, with the aim of achieving success in the care inherent to the maintenance of POD with BD.

As the research continues, the third stage of the methodological approach is planned, which is implementation. Implementation will consist of the use of EMPDO website by a group of nurses, including the collection of information from its use and analysis of results of its usability.

## Data Availability

The research data are available within the article.
