# Supplementary material for: Nursing care in potential organ donor maintenance: EMPDO website validity
Source: Rev Bras Enferm. 2025 Dec 8;78(6):e20240515. doi: 10.1590/0034-7167-2024-0515 (PMC12707527; doi:10.1590/0034-7167-2024-0515)
Supplement: Translation

# Translation: Cuidados de enfermagem na manutenção do potencial doador de órgãos: validação do EMPDO *website*

**DOI:** 10.1590/0034-7167-2024-0515pt

**Authors:** Cláudia Moura Santiago, Cleisiane Xavier Diniz, Maria de Nazaré de Souza Ribeiro, Tereza Cristina Felippe Guimarães, Amélia Nunes Sicsú, Fátima Helena Espírito Santo

**Affiliations:** I Universidade do Estado do Amazonas. Manaus, Amazonas, Brasil; II Instituto Nacional de Cardiologia. Rio de Janeiro, Rio de Janeiro, Brasil; III Universidade Federal Fluminense. Niterói, Rio de Janeiro, Brasil

**Keywords:** Cuidados de Enfermagem, Doação de Órgão, Transplante, Tecnologia em Saúde, *Website*.

## Abstract

**Objetivos:**

validar tecnologia cuidativo-educacional, do tipo *website*, com objetivo de subsidiar a assistência de enfermagem na manutenção do potencial doador de órgãos em morte encefálica.

**Métodos:**

estudo de abordagem metodológica, para validação técnico-científica e didático-ilustrativa do EMPDO *website*. Na dimensão técnico-científica, enfermeiros avaliaram os domínios “objetivos”, “relevância”, “estrutura e apresentação”, utilizando-se o Índice de Validade de Conteúdo. Na dimensão didático-ilustrativa, *web designers* avaliaram os domínios “conteúdo”, “linguagem”, “ilustrações gráficas”, “adequação cultural” e “motivação”. Utilizou-se o ponto de corte do *Suitability Assessment of Materials*.

**Resultados:**

o Índice de Validade de Conteúdo geral do conteúdo técnico-científico atingiu 0,97. Na dimensão didático-ilustrativa, alcançaram-se 25,7 pontos de 26 pontos. As sugestões permitiram corrigir inconsistências para a versão final.

**Conclusões:**

o *website*, construído com as melhores evidências científicas dos cuidados de enfermagem na manutenção do potencial doador em morte encefálica, obteve parecer favorável pelos especialistas, que enfatizaram ser uma tecnologia inovadora, inédita e relevante.

## INTRODUÇÃO

Os processos de doação e transplante de órgãos vêm passando por transformações e avanços científicos que buscam promover a melhora da qualidade e perspectiva de vida dos indivíduos, por meio de capacitações da equipe de saúde responsável e de atualizações baseadas em evidências científicas atuais e inovadoras^(1)^.

Apesar da evolução técnico-científica e da padronização dos cuidados ao potencial doador (PD) de órgãos em morte encefálica (ME), ainda existem empecilhos que resultam na fragmentação da assistência. Alguns incluem o despreparo da equipe na entrevista familiar, a falta de conhecimentos teórico-práticos na manutenção hemodinâmica do paciente, a não realização ou o atraso na abertura do protocolo de ME nas unidades, e a falta de infraestrutura dos hospitais^(2)^.

No que tange ao enfermeiro, este ocupa um lugar de grande relevância na equipe de transplante e doação de órgãos; por isso, precisa estar capacitado e treinado para diversas tarefas, que perpassam a identificação, notificação do doador à equipe de coordenação intra-hospitalar de doação, monitorização e manutenção do PD. Destacam-se também o acolhimento e empatia com a família do PD, o que impactará diretamente a efetivação da captação de órgãos^(1,3-6)^.

Ao se inserir na cultura dos transplantes de órgãos, o enfermeiro passa a compreender o processo do cuidar do PD, na gerência do cuidado, que se inicia com o acolhimento da família, a manutenção do PD e o encaminhamento do doador elegível, encerrando com o retorno do corpo à família^(5)^, tornando os enfermeiros os principais facilitadores do processo de doação de órgãos. Seu conhecimento e atitude em relação à doação de órgãos podem influenciar a opinião pública e de familiares sobre a doação de órgãos^(6)^. Em relação a isso, os resultados de estudo mostraram que 69% das doações de órgãos para transplante ocorreram por intervenções de enfermeiros. Fatores como religião, educação, experiência de trabalho, idade, relacionamento com a família do falecido, história de cuidado de pacientes com morte cerebral e experiências pessoais impactam as atitudes dos enfermeiros diante da doação de órgãos^(7)^.

No Brasil, pesquisadores destacam a importância dos enfermeiros para o sucesso do processo de doação de órgãos, porém identificam também várias desconformidades no que se refere ao conhecimento e aos sentimentos que surgem no cuidado ao paciente PD^(8,9)^. Alguns enfermeiros, ao lidar com o paciente PD em ME, identificam a importância da manutenção do cuidado, porém outros afirmam não priorizar o paciente PD em ME por ele não possuir viabilidade vital. Assim, consideram mais importante cuidar daqueles com prognóstico de vida, negligenciando os devidos cuidados para a manutenção do PD^(9)^.

Estudo transversal realizado com 377 profissionais de saúde em Teerã^(10)^ mostrou que a disposição e a relutância em doar órgãos foram de 47,48% (n=179) e 52,51% (n=198), respectivamente, entre esses profissionais. Na Irlanda, enfermeiros declararam não estar cientes do processo de doação de órgãos e do diagnóstico de ME^(11)^. Na Coréia do Sul, em pesquisa realizada com estudantes de enfermagem, 85,1% informaram não ter recebido nenhuma formação sobre doação de órgãos em ME; 68% estavam dispostos a doar seus órgãos se eles tivessem ME; e 39,5% estavam dispostos a doar os órgãos de familiares sob essas condições^(12)^. Na Índia, menos da metade dos estudantes da área da saúde, que participaram de pesquisa, tinha conhecimento adequado sobre ME e sobre legislação relativa à doação de órgãos^(4)^.

Existe uma certa barreira para se confrontar com a ideia de finitude enquanto se está imerso nas atividades cotidianas^(13)^. Esse pode ser um dos maiores motivos para que o tema da doação de órgãos não esteja na pauta das discussões na sociedade, enquanto as estatísticas continuam a apontar a perda considerável de PD em todo o mundo^(13)^. Somam-se a isso as várias questões relacionadas à fragilidade da equipe de saúde e à manutenção do PD em ME, evidenciando ser imprescindível a elaboração de estratégias educativas voltadas à equipe de saúde, principalmente à equipe de enfermagem^(1)^.

Nesse sentido, os avanços tecnológicos têm favorecido o aprimoramento da assistência no âmbito da saúde de forma eficaz e segura. As tecnologias em saúde têm expressado novas formas de produzi-las, ancoradas na criatividade e desenvolvidas a partir de demandas da realidade^(14)^. Os recursos educacionais no formato digital como *websites* são ferramentas importantes no processo de ensino-aprendizagem, pois auxiliam no aprimoramento do conhecimento de situações vivenciadas em momentos oportunos^(15)^.

As Tecnologias de Informação e Comunicação (TICs) contribuem para o desenvolvimento científico e tecnológico na área da saúde, proporcionando agilidade ao processamento das informações e ampliação da interação do profissional com o usuário^(16)^, promovendo soluções tecnológicas que subsidiam tomada de decisões clínicas, interações e condutas terapêuticas qualificadas^(17)^. Em vista das múltiplas vantagens das TICs, construiu-se uma tecnologia cuidativo-educativa, denominado EMPDO *website*, para nortear a assistência de enfermagem na manutenção do PD de órgãos em ME.

O EMPDO *website* foi lançado em 2023, e consiste em uma tecnologia cuidativo-educativa desenvolvida no âmbito do programa de mestrado profissional em enfermagem em saúde pública da Universidade do Estado do Amazonas. Com a identificação das melhores evidências técnicas, científicas e normativas relacionadas aos cuidados de enfermagem na manutenção do PD de órgãos em ME, a partir de uma *scoping review*, somando a isso a prática profissional das pesquisadoras, foram elencados os conteúdos, organizados sobre os seguintes tópicos que compuseram o *website: Home*; Sobre o EMPDO; Doação de órgãos; Práticas assistenciais nos cuidados ao PD; Materiais exclusivos; Legislação; Boletins; e Notícias. A ferramenta encontra-se disponível no endereço eletrônico https://www.empdo.com.br, e pode ser acessada também por um *QR Code*.

O EMPDO *website* foi criado com rigor científico e um olhar atento sobre as práticas relacionadas à doação de órgãos, com o objetivo de promover um cuidado holístico, almejando resultados favoráveis na preservação do PD em estado de ME. Contudo, uma tecnologia, para ser aplicável, precisa ser validada quanto à sua eficiência e aplicabilidade, comprovando se ela cumpre o propósito para o qual foi criada^(18,19)^.

## OBJETIVOS

Validar tecnologia cuidativo-educacional, do tipo *website*, com objetivo de subsidiar a assistência de enfermagem na manutenção do PD de órgãos em ME.

## MÉTODOS

### Aspectos éticos

A pesquisa foi aprovada pelo Comitê de Ética em Pesquisa da Universidade Estadual do Amazonas. Como garantia de anonimato dos especialistas participantes, quando referenciados no texto, todos receberam uma letra e um número em referência à categoria, seguido da ordem cronológica de resposta ao formulário: Enfermeiro (E1, …); *Designer* (D1, …). Todos assinaram o Termo de Consentimento Livre e Esclarecido (TCLE).

### Desenho, período e local do estudo

Trata-se de estudo de abordagem metodológica e produção tecnológica, que correspondeu à validação, por especialistas da área da saúde (enfermeiros) e *web designer*, de um *website* denominado EMPDO *website*.

Para a definição do tamanho da amostra dos enfermeiros especialistas que validaram a dimensão técnico-científica, foi utilizada a fórmula proposta por Interaminense (2020): n= Zα2. P (1-P) /d2. Onde: Zα2 = 1,96; P = 0,85; d= 0,15. Obteve-se como resultado um total de 22 especialistas, número considerado adequado para uma avaliação com proporção ideal de 85% de aceitação entre os avaliadores, com proporção mínima de 70%, considerando 95% de Intervalo de Confiança^(20)^.

Para o alcance do número adequado de sujeitos da amostra, buscou-se um número de 30 especialistas, mantendo margem de segurança de oito especialistas a mais. Entre os 30 que receberam o convite, 28 avaliaram e validaram o conteúdo da tecnologia proposta.

Para a amostra dos especialistas da dimensão didático-ilustrativa (*designer* gráfico/*web designer*), foram selecionados sete especialistas. Pesquisas apontam que a média ideal seja entre três e cinco participantes^(21-23)^.

### População ou amostra; critérios de inclusão e exclusão

A seleção de todos os especialistas foi realizada de forma não probabilística e intencional, em consulta aos currículos disponíveis na Plataforma *Lattes*, utilizando a ferramenta de busca por assunto, obedecendo aos critérios de inclusão determinados.

Os especialistas da área da saúde, enfermeiros, incluídos na pesquisa, precisaram atender no mínimo a dois dos critérios de inclusão: ter no mínimo três anos de experiência em Unidade de Tratamento Intensivo e manutenção de PD ou áreas afins; ter publicação na área de transplante e/ou manutenção de PD; ter mestrado ou doutorado com produção científica na área de cuidados intensivos ou produção de tecnologias cuidativo-educativa ou em áreas afins; fazer parte de equipe multiprofissional de transplante de órgão há pelo menos um ano.

Com relação aos especialistas da dimensão didático-ilustrativa (*designer* gráfico/*web designer*), foram exigidos dois dos seguintes critérios: experiência profissional com o formato/modalidade de tecnologias cuidativo-educativa há pelo menos dois anos; ter no mínimo três anos de experiência em sua área de atuação; ter produção na área de tecnologias cuidativo-educativas; ser especialista *lato* ou *stricto sensu* na área profissional de *web designer* ou *designer* gráfico.

Como critérios de exclusão para todos, foi considerado inelegível aquele que permaneceu sem dar resposta até o 15º dia após aceitação do convite, comunicou à coordenação do projeto manifestação de sofrimento psíquico durante o processo avaliativo e apresentou dificuldade de compreensão inerente ao método.

Os especialistas de ambas as áreas receberam carta convite mediante contato eletrônico (e-mail via Plataforma *Lattes*). Em seguida, foram enviados o *link* da ferramenta *Google Forms* ^®^, o *link* do *site* para avaliação, o TCLE específico para cada painel de avaliadores, os instrumentos de avaliação e as devidas informações quanto ao preenchimento dos documentos.

### Protocolo do estudo e análise dos resultados

Para coleta dos dados da avaliação da dimensão técnico-científica, utilizaram-se os parâmetros do instrumento de validação adaptado, composto por três domínios (objetivos, relevância, estrutura e apresentação) e 17 itens com valoração de 1 a 4, sendo: 4 - totalmente adequado; 3 - adequado; 2 - parcialmente adequado; e 1 - inadequado. Foi acrescido espaço às sugestões dos especialistas. Para estes dados, foi aplicado o Índice de Validade do Conteúdo (IVC), que avalia a concordância dos especialistas quanto à representatividade da medida em relação ao conteúdo abordado^(24,25)^.

Para o cálculo por domínio, somaram-se os itens avaliados como totalmente adequado e adequado, divididos pelo número total de respostas do domínio. O valor global foi obtido com a média aritmética do IVC por domínio, com parâmetro mínimo de validação pelo IVC de 0,70^(23,26)^. O percentual de concordância que valida um instrumento pode variar de 70% a 100%^(27)^.

Na coleta de dados da dimensão didático-ilustrativa, o instrumento utilizado foi o *Suitability Assessment of Materials* (SAM), adaptado de Galdino (2014), que avalia a tecnologia em cinco domínios: conteúdo; linguagem; ilustrações gráficas; adequação cultural; e motivação^(28)^. Possui 13 itens avaliativos, com valoração de 0 a 2 (0 - inadequado; 1 - parcialmente adequado; e 2 - adequado), para obtenção de escore igual ou superior a 10 pontos do total de 26 pontos^(23,29)^.

Os dados foram lançados em planilhas do *Microsoft Excel^®^*, com dupla conferência, para evitar erros de alimentação do banco de análise, seguido da sua transferência para o *software* estatístico *Statistical Package for the Social Sciences* ^®^, versão 24.0. Calcularam-se as frequências absolutas e relativas, e as médias.

Todos os apontamentos, opiniões e sugestões descritas pelos especialistas nos espaços de comentários, durante a avaliação da tecnologia, foram analisados. Esta etapa permitiu identificar as inconsistências com relação ao conteúdo do *site* e definir o conteúdo para a segunda versão.

## RESULTADOS

Quanto ao perfil dos especialistas que avaliaram a dimensão técnico-científica, a maioria era mulher (n =23; 82%), com mais de três anos de experiência na área (60,7%) e média de 15,75 anos; 13 (46,4%) possuíam titulação *stricto sensu* (doutorado e mestrado); e 14,2% possuíam produção científica na temática de interesse ou participação em eventos sobre o tema nos últimos cinco anos, respectivamente.

Na Tabela 1, são apresentadas as perguntas sobre o conteúdo do *website* com suas respectivas médias ponderadas (IVC por domínio). Ressalta-se que, para avaliar cada item, utilizaram-se os parâmetros do instrumento de validação adaptado, composto por três domínios (objetivos; estrutura e apresentação; e relevância). A análise das respostas foi realizada com o cálculo do IVC por domínio e, posteriormente, pelo valor global. O parâmetro de validação foi de 0,70 de IVC.

**Tabela 1:** Distribuição dos escores de concordância e do Índice de Validade de Conteúdo segundo os itens por domínio obtidos das respostas dos juízes especialistas da dimensão técnico-científica do EMPDO *website*, 2023

1 - OBJETIVOS - Referem-se a propósitos, metas ou fins que se deseja atingir com a utilização da tecnologia.							
**Itens do domínio**		**TA**	**A**	**PA**	**I**	**TA+A**	**IVC**
1	As informações e/ou conteúdos são ou estão coerentes com as necessidades cotidianas do público-alvo da tecnologia.	15	13	0	0	28	1,0
2	As informações e/ou conteúdos são importantes para a qualidade do cuidado ou o trabalho do público-alvo da tecnologia.	15	13	0	0	28	1,0
3	Convida e/ou instiga a mudanças de cuidados na manutenção do potencial doador de órgãos pelos enfermeiros.	11	16	1	0	27	0,96
4	Pode circular no meio científico da área (saúde).	19	9	0	0	28	1,0
5	Atende aos objetivos da pesquisa e de instituições que atendem/trabalham com o público-alvo da tecnologia.	16	12	0	0	28	1,0
**IVC domínio 1 (objetivos): 0,99**							
**2- ESTRUTURA E APRESENTAÇÃO - Refere-se à forma de apresentar as orientações. Isso inclui organização geral, estrutura, estratégia de apresentação, coerência e formatação.**							
**Itens do domínio**		**TA**	**A**	**PA**	**I**	**TA+A**	**IVC**
1	A tecnologia é apropriada para o público-alvo.	13	15	0	0	28	1,0
2	Os conteúdos estão apresentados de maneira clara e objetiva.	16	11	1	0	27	0,96
3	As informações apresentadas estão cientificamente corretas.	14	13	1	0	27	0,96
4	O material está apropriado ao nível do público-alvo.	9	18	1	0	27	0,96
5	Há uma sequência lógica do conteúdo proposto.	14	14	0	0	28	1,0
6	As informações estão bem estruturadas em concordância e ortografia.	11	16	1	0	27	0,96
7	O texto está claramente descrito e/ou sintetizado, respondendo às necessidades do público-alvo.	10	16	2	0	26	0,92
**IVC domínio 2 (estrutura e apresentação): 0,96**							
**3 RELEVÂNCIA - Refere-se às características que avaliam o grau de significância da tecnologia.**							
**Itens do domínio**		**TA**	**A**	**PA**	**I**	**TA+A**	**IVC**
1	Os temas retratam aspectos-chave que devem ser reforçados.	15	13	0	0	28	1,0
2	A tecnologia permite a generalização e transferência do aprendizado a diferentes contextos.	12	15	1	0	27	0,96
3	A tecnologia propõe a construção de conhecimentos.	17	11	0	0	28	1,0
4	A tecnologia aborda os assuntos necessários para o saber do público-alvo.	12	16	0	0	28	1,0
5	A tecnologia está adequada para ser usada por qualquer profissional com o público-alvo.	15	12	1	0	27	0,96
**IVC domínio 3 (relevância): 0,98**							
Domínio 1 - objetivos/domínio 2 - estrutura e apresentação/domínio 3 - relevância **IVC GERAL:** domínio 1 (0,99) + domínio 2 (0,96) + domínio 3 (0,98) --------------------------------------------------------------------------------------------------- / 3 = **0,97**							

*IVC - Índice de Validade de Conteúdo; TA - totalmente adequado; A - adequado; PA - parcialmente adequado; I - inadequado.*

O Quadro 1 sintetiza as avaliações de especialistas sobre o *website* EMPDO, abrangendo os domínios “objetivos”, “estrutura e apresentação” e “relevância”. Na aba de observações, foram feitas algumas sugestões pelos participantes da pesquisa, que foram analisadas e acatadas, de forma a aprimorar o conteúdo e a visualização do *website*.

**Quadro 1:** Considerações dos especialistas enfermeiros relativos aos domínios “objetivos, “estrutura e apresentação” e “relevância”, e respostas das pesquisadoras sobre o EMPDO website

Domínio objetivos	
**Considerações dos especialistas**	**Considerações da pesquisadora**
E2, E3, E10, E25 - Sugerem revisão da Sistematização de Assistência de Enfermagem, pois alguns diagnósticos não se encaixam e há textos que não foram mencionados no Processo de Enfermagem (etapas).	Foi ajustado o Processo de Enfermagem com todas as etapas descritas e elencadas (coleta de dados, diagnósticos (NANDA)/planejamento (NOC)/intervenções (NIC), avaliação, outros cuidados e estratégias de enfermagem).
E3, E9, E11, E20 - Esperam que o site se mantenha atualizado anualmente.	Estão programadas atualizações semestrais com ajuda de docentes e discentes em parceria com a Universidade do Estado do Amazonas.
E16 - Sugere mudança na nomenclatura e sigla de potencial doador de órgãos para PD.	Retificou-se a sigla para PD - potencial doador.
E12, E21, E22, E28 - Sugerem acrescentar sugestões, comentários e filmes.	Criaram-se duas páginas contemplando as possibilidades de comentários e sugestões, filmes e outros.
**Domínio estrutura e apresentação**	
**Considerações dos especialistas**	**Considerações da pesquisadora**
E2 - Sugere, na página principal, retificar a concordância numeral de “enfermeiros”.	Corrigido, conforme apontado pelo E2.
E5, E6, E10 - Sugerem que o texto passe por uma revisão do português, revisão ortografia, formatação e acentuação.	O texto foi revisado.
E14 - Sugere que seria interessante acrescentar um “algoritmo de manejo do paciente potencial doador”.	Foram acrescentados alguns algoritmos em destaque que já estão sendo utilizados.
E16 - Sugere criar *QR Code* para divulgação.	Foi criado *QR Code* para divulgação.
E15 - Sugere trocar para “existem dois tipos de doadores: vivos ou falecidos (cadáveres)”.	Foi acrescentada a palavra “cadáveres”.
E17, E12, E13, E18, E20 - Sugerem eventos para divulgação do *website* e para profissionais especialistas na área.	Foi criada uma agenda de divulgação em eventos pertinentes à área da enfermagem.
E28 - Sugere que os cuidados com as córneas precisam ser revistos e mais bem esclarecidos.	Acrescidos e melhorados conteúdos sobre os cuidados pertinentes à doação de córnea.
E21 - Sugere elaboração de artigos e registro (inserção) do *site*.	Acatada a sugestão, com inclusão de diversos artigos sobre o tema no *site*. Os artigos elaborados pelas autoras, após sua devida publicação, serão inseridos no *site*.
**Domínio relevância**	
**Considerações dos especialistas**	**Considerações da pesquisadora**
E6 - Sugere que, pela relevância que tem, poderia possuir aba de acesso à programação de eventos/cursos nacionais e/ou internacionais, e contatos das Centrais Estaduais de Transplantes.	Foram acrescidos os contatos das Centrais Estaduais de Transplantes e, na alimentação do site, serão inseridos eventos relativos ao tema.
E10 - Sugere que, em algumas páginas, as imagens não apresentam nitidez. Para que tenha relevância, é preciso melhorá-las.	As imagens foram melhoradas quanto à sua nitidez.

### Validação da dimensão didático-ilustrativa/aparência do EMPDO *website* pelos *designer* gráficos/*web designers*

Sete *web designers* responderam à avaliação da dimensão didático-ilustrativa/aparência. Cada um recebeu uma identificação fictícia, representada pela letra D (*designer*) e números de 1 a 7, de modo a preservar sua identidade. Entre os que avaliaram a tecnologia, 57% eram homens; 57% tinham titulação *stricto sensu;* 100% tinham tempo de formação superior a sete anos; todos tinham acima de quatro anos de experiência.

Para a avaliação da dimensão didático-ilustrativo/aparência, foi utilizado o SAM, com cinco dimensões, nomeadamente “conteúdo”, “linguagem”, “ilustrações gráficas”, “motivação” e “adequação cultural”, com um total de 13 itens, com ponto de corte mínimo de 10 pontos e máximo de 26 pontos, com valoração de 0 a 2 para cada item, sendo: 2 - se concordassem que o *website* é adequado; 1 - parcialmente adequado; e 0 - inadequado^(23,29)^.

A pontuação geral atingiu 25,7 pontos, com pontuação máxima de 26 pontos, julgando o *site* adequado pelos *web designers/designers* gráficos. A avaliação teve variância de 25 a 26 pontos, com dois especialistas avaliando o material com 25 pontos (Quadro 2).

**Quadro 2:** Pontuação individual dos *web designers* **por domínio obtidos das respostas sobre a dimensão técnico-científica do EMPDO** *website*, 2023

ESPECIALISTAS	PONTUAÇÃO POR DOMÍNIO					
	Conteúdo	Linguagem	Ilustração gráfica	Motivação	Adequação cultural	Pontuação final
D1	6	5	4	6	4	25
D2	6	6	4	6	4	26
D3	6	6	4	6	4	26
D4	6	6	3	6	4	25
D5	6	6	4	6	4	26
D6	6	6	4	6	4	26
D7	6	6	4	6	4	26
**Pontuação média**		Pontuação por especialistas/total especialistas 25+26+26+25+26+26+26 / 7 **Pontuação média = 25,7 pontos**				

Com relação à pontuação atribuída por domínio, o Quadro 3 mostra que os domínios “conteúdo”, “motivação” e “adequação cultural” obtiveram pontuação máxima em todos os seus itens avaliados, alcançando 100%. O cálculo geral foi feito pela multiplicação das respostas dos sete especialistas, atingindo 180 pontos (98,9%) de um total geral de 182 pontos (100%) (Quadro 3).

**Quadro 3:** Distribuição dos pontos com cada *web designer* **de acordo com as respostas obtidas em cada item segundo conteúdo, linguagem, ilustrações gráficas, motivação e adequação cultural do EMPDO** *website*, 2023

Itens do domínio		A 02	PA 01	I 00	Pontos obtidos
**Domínio 1 - CONTEÚDO**					
1.1	O objetivo é evidente, facilitando a pronta compreensão do *website*.	07	-	-	14
1.2	O conteúdo aborda informações relacionadas aos cuidados de enfermagem na manutenção do potencial doador de órgãos.	07	-	-	14
1.3	A proposta do *website* é interessante e inovadora, permitindo o público-alvo acessar conteúdos sobre a temática em questão.	07	-	-	14
	**Domínio 1 - Conteúdo**	**42**			
**Domínio 2 - LINGUAGEM pontos**					
2.1	O nível de leitura é adequado para a compreensão do público-alvo (profissionais de saúde).	07	-	-	14
2.2	O estilo visual é coerente com o conteúdo descrito.	06	-	-	13
2.3	O vocabulário utiliza palavras comuns.	07	-	-	14
	**Domínio 2 - Linguagem**	**41**			
**Domínio 3 - ILUSTRAÇÕES GRÁFICAS**					
3.1	A interface atrai a atenção e retrata o propósito do *website*.	07	-	-	14
3.2	As ilustrações visuais e descrições apresentam mensagens fundamentais para que o leitor possa compreender os cuidados para manutenção dos potenciais doadores de órgãos.	06	01	-	13
	**Domínio 3 - Ilustrações gráfica**	**27**			
**Domínio 4 - MOTIVAÇÃO**					
4.1	Ocorre interação do texto com o leitor, levando-os a fazer escolhas para manter o potencial doador de órgãos.	07	-	-	14
4.2	Há uma sequência lógica do conteúdo proposto, trazendo a temática em questão.	07	-	-	14
4.3	O *website* está apropriado ao nível do público-alvo.	07	-	-	14
	**Domínio 4 - Motivação**	**42**			
**Domínio 5 - ADEQUAÇÃO CULTURAL**					
5.1	O *website* pode ser usado por profissionais de qualquer região.	07	-	-	14
5.2	Apresenta imagens e exemplos adequados.	07	-	-	14
	**Domínio 5 - Adequação cultural**	**28**			
**PONTUAÇÃO GERAL**		**42 + 41 + 27 + 42 + 28 = 180 pontos** Sete especialistas **25,7 pontos (98,9%)**			

*A - adequado; PA - parcialmente adequado; I - inadequado.*

O site “EMPDO - Enfermagem na Manutenção do Potencial Doador de Órgãos” pode ser acessado diretamente pelo *link* www.empdo.com.br ou através de um *QR Code* (Figura 1). A plataforma oferece uma série de recursos e orientações externas para a prática de enfermagem na manutenção de PD de órgãos, disponibilizando informações atualizadas, manuais técnicos, vídeos explicativos e diretrizes baseadas em evidências científicas. Além disso, o *site* apresenta descrições interativas para consulta de protocolos e acesso materiais exclusivos, facilitando o aprimoramento do conhecimento técnico e o suporte ao cuidado para as práticas de enfermagem ao PD.

**Figura 1:** *QR Code* **do EMPDO** *website*

## DISCUSSÃO

O domínio 1 (objetivos) possui cinco itens e refere-se aos propósitos, metas ou fins que se deseja atingir com a utilização da tecnologia. Dos 28 especialistas, 27 (96,4%) julgaram os itens deste domínio como totalmente adequados e adequados, e apenas um julgou um dos itens como parcialmente adequado. Observa-se, na Tabela 1, que houve concordância, com valoração no IVC de 0,99 para objetivos, do IVC entre todos os domínios, validando o *website* neste item.

No domínio 2 (estrutura e apresentação), avaliaram-se a organização, estrutura, estratégia de apresentação, coerência e formatação do *website* nos sete itens presentes no instrumento. A Tabela 1 mostra que 26 (92,8%) dos especialistas avaliaram a tecnologia nesses itens como totalmente adequada e adequada. Destaca-se que, em alguns itens, a valoração alcançou 100% de respostas para totalmente adequado e adequado. O IVC deste domínio atingiu valoração de 0,96. Apesar de ter alcançado um valor considerado alto de aceitação, houve seis respostas indicando parcialmente adequado, com apontamentos direcionados a erros ortográficos.

No domínio 3 (relevância), avaliou-se o grau de significação da tecnologia em cinco itens, no qual 27 (96,4%) dos especialistas atribuíram valoração de totalmente adequado e parcialmente adequado, com concordância na maioria dos itens. Houve avaliação “parcialmente adequado” em apenas dois itens, atingindo o IVC de 0,98.

Na avaliação do conteúdo do *website* pelos especialistas enfermeiros, todas as perguntas realizadas conseguiram alcançar um IVC acima de 0,90. Sendo assim, não houve necessidade de realizar grandes alterações.

No que tange ao domínio “objetivos”, E1, E5 e E14 citam que o *site* tem excelente apresentação e conteúdo, que o material está muito bem elaborado e atende aos objetivos e metas propostos. E8 cita que “a tecnologia é inovadora e irá facilitar a compreensão dos profissionais mediante o acesso ao conteúdo”. E7, E23 e E24 salientam a relevância e a excelência do *website* e que ele trará contribuição no aperfeiçoamento de profissionais da área da saúde e auxiliará na rotina do plantão de quem trabalha com o PD de órgão.

No que diz respeito ao domínio “estrutura e apresentação”, E1, E4, E7, E9, E14, E23 e E24 destacam a excelência do *website* no que diz respeito à estrutura e apresentação, parabenizando as autoras pelos conteúdos inseridos, apresentados de maneira clara, objetiva, bem descrito e/ou sintetizado, respondendo às necessidades do público-alvo, com conteúdo em forma de texto bem elaborado. Os avaliadores julgam ser uma tecnologia acessível, gratuita e universal aos profissionais de enfermagem. O E8 destaca que “é uma tecnologia que se ajusta à proposta de difundir mais conhecimento ao público em geral, pois ainda há muitas resistências na doação de órgãos, por falta de conhecimento”.

No domínio “relevância”, E1, E14, E23 e E24 descrevem que o *website* é uma ferramenta importante e de excelência, com método claro para o uso dos profissionais na área da saúde. E6 destaca que “a ferramenta é bastante pertinente para melhoria da assistência prestada”. E18 ressalta que “é uma tecnologia que irá ajudar a disseminar mais conhecimentos e permitir generalização e transferência do aprendizado em diferentes contextos”. E21 afirma que “está na forma adequada para ser usada por qualquer profissional de enfermagem e, por isso, tem grande relevância”.

### Validação da dimensão didático-ilustrativa/aparência do EMPDO *website* pel*os designers* gráficos/*web designers*

Os especialistas não fizeram comentários e sugestões; portanto, o *website* não necessitou de alterações nessa dimensão. O *website* foi considerado adequado e pode ser utilizado como estratégia para disseminar o conhecimento e aprimorar as técnicas e habilidades dos enfermeiros na área de transplante e doação de órgãos.

Estudos demonstram que o desenvolvimento de estratégias educativas e tecnológicas com foco na qualificação e capacitação dos profissionais, visando à qualidade do cuidado, é uma necessidade no cenário atual da enfermagem^(15,30)^.

Sendo assim, observa-se que o conhecimento é fundamental para o cuidado ao PD de órgãos, porém, para que não haja déficit e lacunas neste cuidado, devem-se buscar a padronização e uso de protocolos e guias de assistência no cuidado a ser prestado^(1,31)^. No entanto, estudos demonstram que ainda há uma fragilidade no âmbito da prestação de cuidados no processo de doação e transplante de órgãos. A equipe de enfermagem comumente demonstra insegurança acerca do suporte a ser dado ao PD e dúvidas quanto às questões éticas que envolvem esses pacientes^(1,32)^.

Nesse contexto, o EMPDO *website* foi produzido com cientificidade e aprofundamento quanto aos cuidados ao PD de órgãos, no intuito de apontar um cuidado integral, visando resultados positivos na manutenção do PD em ME. Acessando o EMPDO *website*, o usuário encontrará diversos conteúdos científicos que dizem respeito à manutenção do PD de órgãos.

Para garantir a eficiência e a eficácia do Processo de Enfermagem, deu-se destaque ao cuidado do paciente em ME, enfatizando a importância de uma minuciosa avaliação clínica e social. Entre as intervenções, ressaltaram-se o cuidado com a hidratação contínua das córneas, controle dos sinais vitais, realização das dosagens eletrolíticas e gasométricas, hemoculturas e urocultura, verificando as respostas em relação aos resultados esperados.

A construção do *website* durou aproximadamente quatro meses, desde a elaboração de conteúdo até a finalização da estrutura do *site*, totalizando 49 páginas da *web*. Encontra-se implementado com domínio já registrado. Dentro do que se espera de uma tecnologia cuidativo-educacional, o EMPDO *website* fomenta a socialização de informações relevantes que contribuem na assistência de enfermagem para a manutenção do PD de órgãos em ME. Mais do que sua proposta original e conceito, é sempre importante entender o impacto positivo que ele pode ter sobre aqueles que o utilizarem.

### Limitações do estudo

Como limitações do estudo, destacam-se o tempo restrito para aplicação e avaliação de usabilidade do *website*.

Um dos pontos de divergências entre diferentes autores é a quantidade e qualificação dos especialistas envolvidos no processo de validação. Alguns sugerem uma participação que varia de cinco a dez pessoas nesse processo; outros apontam uma faixa de seis a vinte participantes. Ao tomar essa decisão, é fundamental considerar as características do instrumento, assim como a formação, a competência e a disponibilidade dos profissionais requisitados. A amostra de 28 especialistas da área da saúde, determinada para este estudo, proporcionou uma demora para alcançar o número desejado, o que pode ser repensado para próximos estudos utilizando uma amostra quantitativamente menor e qualitativamente melhor.

Embora o SAM seja considerado uma ferramenta útil para avaliar a adequação de materiais educacionais, ele possui limitações significativas; entre elas, considera-se que a avaliação é susceptível a influências subjetivas do avaliador, o que pode levar a variações nos resultados. A personalização para o público-alvo e a compreensão dos dados apresentados são vitais para a eficácia dos materiais, especialmente em saúde e educação. Portanto, apesar de sua relevância, é essencial considerar tais limitações do SAM e buscar abordagens complementares para avaliações mais precisas.

Em relação ao *website*, a necessidade de acompanhar continuamente o avanço acelerado da tecnologia e ajustar a ferramenta em uso requer um investimento e atualização constante. Dificuldades de conexão e outras questões técnicas podem impactar o desempenho da plataforma e motivar seu desuso.

### Contribuições para as áreas da enfermagem, saúde ou políticas públicas

Dentro do que se espera de uma tecnologia cuidativo-educativa, o EMPDO *website* fomenta a socialização de informações relevantes que contribuem na assistência de enfermagem para a manutenção do PD de órgãos em ME.

Recursos interativos, como o EMPDO *website*, tornam o aprendizado mais dinâmico e envolvente, aprimorando habilidades de comunicação e pesquisa essenciais no mundo contemporâneo. O conteúdo disponibilizado pode ser atualizado constantemente, garantindo que todos os interessados tenham acesso a informações recentes na área em questão, promovendo o engajamento do enfermeiro no cuidado ao PD de órgãos.

## CONCLUSÕES

Neste estudo, adotou-se a validação multiprofissional envolvendo enfermeiros e *designers* gráficos. Os primeiros validaram o conteúdo técnico-científico, e os segundos, a dimensão didática-ilustrativa/aparência. Em ambas as categorias, a tecnologia produzida obteve avaliação satisfatória, corroborando para ratificar sua pertinência quanto ao que se propõe.

Na distribuição dos escores de concordância e do IVC, segundo os itens por domínio obtidos das respostas dos enfermeiros especialistas que avaliaram a dimensão técnico-científica do EMPDO *website*, o resultado obtido pelo cálculo do IVC por domínio foi domínio 1 (0,99), domínio 2 (0,96), domínio 3 (0,98). O IVC global foi de 0,97.

Com relação às respostas obtidas dos *web designers*, que avaliaram conteúdo, linguagem, ilustrações gráficas, motivação e adequação cultural, a pontuação geral atingiu 25,7 pontos, do máximo de 26 pontos, o que representa 98,9%.

A validação do EMPDO *website* alcançou um escore acima de 0,90 no IVC específico e geral referente ao conteúdo técnico-científico e na dimensão didático-ilustrativa/aparência, o que significa que o produto foi considerado adequado, obtendo parecer favorável pelos especialistas enfermeiros e *web designers*, que enfatizaram ser uma tecnologia inovadora, inédita e de grande relevância.

As sugestões, embora pontuais, foram acatadas, aperfeiçoando a tecnologia cuidativo-educativa. Espera-se que o EMPDO *website* contribua de forma teórica e prática para os profissionais de enfermagem atuantes no processo de transplante e doação de órgãos, no intuito de lograr êxito quanto aos cuidados inerentes à manutenção do PD de órgãos em ME.

Na continuidade da pesquisa, prevê-se a realização da terceira etapa da abordagem metodológica, que é a implementação. A implementação consistirá no uso do EMPDO *website* por um conjunto de enfermeiros, incluindo a coleta de informações a partir de seu uso e análise dos resultados de sua usabilidade.

## Data Availability

Os dados da pesquisa estão disponíveis no corpo do artigo.
